# Does the IL-6/KL-6 ratio distinguish different phenotypes in COVID-19 Acute Respiratory Distress Syndrome? An observational study stemmed from prospectively derived clinical, biological, and computed tomographic data

**DOI:** 10.1371/journal.pone.0321533

**Published:** 2025-05-21

**Authors:** Nicolas Partouche, Myriam Maumy, Thien-Nga Chamaraux-Tran, Frederic Bertrand, Francis Schneider, Nicolas Meyer, Morgane Solis, Samira Fafi-Kremer, Eric Noll, Julien Pottecher

**Affiliations:** 1 Service d’Anesthésie-Réanimation & Médecine Péri-Opératoire, Hôpital de Hautepierre, Hôpitaux Universitaires de Strasbourg, UR3072, FMTS, FHU Omicare, Faculté de Médecine, Maïeutique et Sciences de la Santé, Université de Strasbourg, Strasbourg, France; 2 LIST3N, University of Technology of Troyes, Troyes, France; 3 Institut de Génétique et de Biologie Moléculaire et Cellulaire (IGBMC), CNRS UMR7104, INSERM U1258, Université de Strasbourg, 1 Rue Laurent Fries, Illkirch-Graffenstaden, France; 4 Service de Médecine Intensive-Réanimation, Hôpital de Hautepierre, Hôpitaux Universitaires de Strasbourg, Strasbourg, France; 5 Service de santé Publique, GMRC, Hôpitaux Universitaires de Strasbourg, Strasbourg, France; 6 Faculté de Médecine, Laboratoire de Virologie, Hôpitaux Universitaires de Strasbourg, Strasbourg, France - INSERM, UMR_S1109, LabEx TRANSPLANTEX, Centre de Recherche d’Immunologie et d’Hématologie, Fédération Hospitalo-Universitaire (FHU) OMICARE, Fédération de Médecine Translationnelle de Strasbourg (FMTS), Université de Strasbourg, Strasbourg, France; Children's National Hospital, George Washington University, UNITED STATES OF AMERICA

## Abstract

**Background:**

As new SARS-CoV-2 variants emerge and as treatment of COVID-19 ARDS remains exclusively supportive, there is an unmet need to better characterize its different phenotypes to tailor personalized treatments. Clinical, biological, spirometric and CT data hardly allow deciphering of Heavy (H), Intermediate (I) and Light (L) phenotypes of COVID-19 ARDS and the implementation of tailored specific strategies (prone positioning, PEEP settings, recruitment maneuvers). We hypothesized that the ratio of two pivotal COVID-19 biomarkers (interleukin 6 [IL-6] and Krebs von den Lungen 6 [KL-6], related to inflammation and pneumocyte repair, respectively) would provide a biologic insight into the disease timeline allowing 1) to differentiate H, I and L phenotypes, 2) to predict outcome and 3) to reflect some of CT findings.

**Methods and findings:**

This was a retrospective analysis of prospectively acquired data (COVID HUS cohort). Inclusion concerned any patient with severe COVID-19 pneumonia admitted to two intensive care units between March 1^st^ and May 1^st^, 2020, in a high-density cluster of the first epidemic wave (Strasbourg University Hospital, France). Demographic, clinical, biological (standard, IL-6 [new generation ELISA], KL-6 [CLEIA technique]), spirometric (driving pressure, respiratory system compliance) and CT data were collected longitudinally. CT analysis included semi-automatic and automatic lung measurements and allowed segmentation of lung volumes into 4 (poorly aerated, non-aerated, overinflated and normally aerated) and 3 (ground-glass, restricted normally aerated, and overinflated) zones, respectively. The primary outcome was to challenge the IL-6/KL-6 ratio capacity to decipher the three COVID-19 ARDS phenotypes (H, I and L) defined on clinical, spirometric and radiologic grounds. Secondary outcomes were the analysis of the prognostic value of the IL-6/KL-6 ratio and its correlates with CT-acquired data. Multivariate analysis was based on principal component analysis.

One hundred and forty-eight ventilated COVID-19 ICU patients from the COVID HUS cohort were assessed for eligibility and 77 were included in the full analysis. Most were male, all were under invasive mechanical ventilation and vasopressor therapy and displayed high severity scores (SAPSII: 48 [42–56]; SOFA: 8 [7–10]). The L, I and H COVID ARDS phenotypes were identified in 11, 15 and 48 patients, respectively. In three patients, the phenotype could not be defined precisely. Thirty patients (39%) died in the ICU and the number of ventilator-free days was 2 [0–2] days. The IL-6/KL-6 ratio was not significantly different between the L, I and H phenotypes and evolved according to similar patterns over time. Surviving and deceased patients displayed an inverse kinetic of KL-6. IL-6 and the IL-6/KL-6 ratio were linearly associated with ground-glass volume on semi-automatic and automatic CT lung measurements.

**Conclusions:**

In our population of severe ventilated COVID ARDS patients, the IL-6/KL-6 ratio was not clue to differentiate the H, I and L phenotypes and tailor a personalized ventilatory approach. There was an interesting correlation between IL-6/KL-6 ratio and ground-glass volume as determined by automated lung CT analysis. Such correlation deserves more in-depth pathophysiological study, at best gathered from a prospective cohort with a larger sample size and histological analysis.

**Trial registration:**

COVID HUS Trial registration number: NCT04405726

## Introduction

In December 2019, the city of Wuhan became the epicenter of a viral pneumonia named Coronavirus disease 2019 (COVID-19) [1], caused by a new virus of the coronavirus family, called “severe acute respiratory syndrome coronavirus 2” (SARS-CoV-2). In a short time, this pathology became a pandemic which considerably affected health systems worldwide and imposed drastic restrictions that profoundly altered the professional and social life of humanity.

As of March 3, 2024, COVID-19 has resulted in over 774 million confirmed cases and over 7 million deaths worldwide [1]. COVID-19 patients admitted to the intensive care unit (ICU) have a high mortality rate [2]. The COVID-ICU Group’s prospective study conducted in French, Belgian and Swiss hospitals in Europe during the first wave of the COVID-19 epidemic showed that 35% of patients admitted to the ICU died during their ICU stay [3]. The improvement of our knowledge of SARS-CoV-2, and the introduction of therapies have led to a reduction in ICU mortality, with a death rate of around 20% at the end of this first wave, regardless of age, gender, or comorbidities.[4]

Currently, there are treatments that can prevent SARS-CoV-2 infection or its progression to a severe form of COVID-19 (vaccine, corticosteroid therapy, anticoagulation, monoclonal antibodies), but none of them can either cure full-blown severe COVID or eradicate SARS-CoV-2 from the face of the earth. This, together with numerous mutations in SARS-CoV-2 which are responsible for new and potentially more contagious variants, explains the genesis of several successive epidemic waves, highlighting the need to improve our understanding of the pathogenicity of severe forms of COVID-19 to better manage patients.

It appears that the pattern of COVID-19 pneumonia is progressive and variable depending on the time elapsed since primary infection, the patient’s ventilatory responsiveness to hypoxemia, and host response.[5] Indeed, Gattinoni *et al*. argue that the interaction between these factors leads to the development of a time-related spectrum of diseases within two main “phenotypes”: type L (light), and type H (heavy) phenotypes characterized by clinical, spirometric and radiological criteria. This phenotypic classification mainly relies on static respiratory compliance values measured at the bedside. It should be noted that the classification of L and H phenotypes proposed by Gattinoni et al.[5] has given rise to much discussion in the medical literature concerning its validity and clinical usefulness in the management of COVID-19 pneumonia. In several articles published in the *European Respiratory Journal* [6,7], researchers have raised concerns about the simplistic and rigid nature of this categorization. It has been suggested that the progression between these phenotypes is more fluid than the initial article suggests. Other studies have highlighted the complexity of inflammatory responses and underlying mechanisms in COVID-19, making it difficult to reduce patients to two distinct categories [8]. For instance, in their Letter to the Editor, Gattinoni, Camparota and Marini admit that the L/H classification is primarily theoretical and intended to guide respiratory therapeutics, and that it must be interpreted with caution [9]. Other critics point out that parameters such as inflammatory status and early management of hypoxemia play a major role and cannot be neglected, thus complicating clinical management based solely on these phenotypes. Finally, alongside these two main phenotypes, an intermediate phenotype [10] (phenotype I) refers to a subgroup of patients with compliance characteristics that do not correspond to phenotypes L and H.

In addition to this clinical presentation, the anatomical-pathological data [11] also suggest that both phenotypes may coexist. Initial autopsy findings revealed a lymphocytic viral pneumonia corresponding to an inflammatory infiltrate of the pulmonary tissue that could be considered as type L. Conversely, recently gathered pathologic specimens showed another histological signature called acute fibrinous and organizing pneumonia (AFOP). AFOP is characterized by extensive intra-alveolar fibrin balls, embedded by fibroblasts, type 2 pneumocyte hyperplasia and without hyaline membrane deposition, which could be considered as type H. Further autopsy findings revealed distinctive vascular features such as initial injury to the pulmonary endothelium, intussusceptive angiogenesis, widespread thrombosis, impairment of hypoxic pulmonary vasoconstriction (both contributing to intrapulmonary shunt) and limited epithelial damage [12].

Some drug strategies are effective at an early stage, when viral replication is active and inflammatory processes are at the forefront, but are not useful or even potentially harmful at a more advanced stage of the disease when other therapies may be more advisable. Similarly, the ventilatory strategy must be adapted to the different stages of alveolar damage (L phenotype versus H phenotype), which are difficult to identify based on clinical, spirometric and scanographic criteria alone [13,14]. Indeed, the compliance of the respiratory system (C_RS_) can only be measured in ventilated patients. With successive waves, less and less severe ICU COVID-19 patients were invasively ventilated, and many were maintained on non-invasive support (high-flow nasal oxygenation or non-invasive mask ventilation with pressure support). Second, measured C_RS_ is intricately linked to extent of the aerated lung at the time of measurement, which largely depends on the timing of endotracheal intubation and invasive mechanical ventilation. Third, accurate measurement of C_RS_ requires an end-inspiratory hold manoeuvre during volume-controlled continuous mandatory ventilation with constant flow. Such manoeuvre reveals easy in patients passively ventilated under muscle relaxants but may prove difficult and provide inaccurate measurement of C_RS_ in patients with respiratory efforts and increased inspiratory “drive”. This is true even with new generation ventilators using the linear least squares fitting method for the equation of motion [15]. We and others have observed that severe COVID-19 patients regularly displayed a large respiratory drive [16] and profound inspiratory effort, even on mechanical ventilation and sedation when they were not given muscle relaxants. Such conditions may impede accurate measurements of C_RS_.

Thus, we believe that biomarkers would facilitate the differentiation of the H, I and L phenotypes when C_RS_ measurement is not possible or flawed and allow for an individualized approach of the severe COVID-19 ARDS patient. Indeed, use of standard positive end-expiratory pressure/FiO_2_ tables, higher positive end-expiratory pressure strategies, and higher tidal volumes may all be potentially deleterious in L phenotype [17]. So, we wondered whether biological criteria could possibly distinguish these two phenotypes, to allow a tailored management of ventilated ICU patients with a severe form of COVID-19-associated ARDS (CARDS). Based on current knowledge, we challenged the ratio of two COVID-19 pivotal biomarkers (interleukin 6 and Krebs von den Lungen 6 protein) to match this goal.

Early clinical studies in China have reported an association between SARS-CoV-2-induced hyperinflammation syndrome and disease severity [18–20]. This hyperinflammatory state is called “cytokine storm”. Cytokine storm” is characterized by an increased level of pro-inflammatory cytokines exacerbating inflammation and contributing to tissue injury. Among all cytokines released in the COVID-19 cytokine storm, interleukin 1 (IL-1), interleukin 6 (IL-6), TNF-α and IFN-γ are prominent. TNF-α is a major player in the induction of systemic inflammation and the destruction of healthy cells [21]. Similarly, IFN-γ, which is involved in the antiviral immune response, may trigger an amplification of the inflammatory response when released in excess, aggravating lung lesions [22].

According to Liu and Hill [21], IL-6 plays a central role in the worsening of the inflammatory response and is strongly associated with the severity of symptoms, particularly in acute respiratory distress syndrome. This is interesting because IL-6 was found to be strongly associated with COVID-19 outcome [23–25]. It has therefore been hypothesized that the rate of IL-6 release could be high, and probably more in the early phase of the disease and in the L phenotype because the latter is contemporary to an inflammatory profile.

On the other hand, it was seen earlier that the histological appearance that corresponds to the H phenotype is AFOP with pneumocyte 2 hyperplasia. Krebs von den Lungen 6 protein (KL-6) is a glycoprotein expressed predominantly by type 2 pneumocytes and expressed more prominently during proliferation of these cells [26,27]. KL-6 has been shown to be higher in patients with severe COVID-19, who are more likely to progress to an H phenotype, than those with mild to moderate COVID-19 [28]. So, KL-6 may be a promising biomarker of interest for the H phenotype.

Taken as a whole, IL-6 and KL-6 are two prognostic biomarkers whose concentrations kinetics, taken independently, seem to evolve in an opposite way over the course of COVID-19 and among the two prevailing phenotypes. In the clinical arena, the IL-6/KL-6 ratio may prove useful as 1) the accurate determination of the L and H phenotypes (or the transition from L- to H-type within a same patient) requires extensive reiterated, high-end spirometric and computed tomography analysis [29] and 2) treatments tailored to one or the other phenotype may improve care and outcome [30–32] even if the existence of different phenotypes is still debated [6,9]. Phenotyping COVID-ARDS [33] but also ARDS from other causes than SARS-CoV-2 infection [34,35] was recently determined a promising way for personalized precision medicine.

Confronting the ratio of these two biomarkers with a set of clinical, CT-scan and spirometric data could allow us to determine whether the IL-6/KL-6 ratio is a good candidate to better identify the phenotypic profiles of our patients with severe COVID-19. The aim of our study was therefore to provide full clinical, spirometric and CT data of severe ICU COVID-19 patients hospitalized in a high-density cluster of the first wave (alpha variant) and confront them to the IL-6/KL-6 ratio to challenge its ability to adequately distinguish COVID-19 ARDS phenotypes.

## Methods

### 1. Study design and participants

In this retrospective, single-center dual site study, all adult patients (aged ≥18 years) who had a confirmed diagnosis of SARS-CoV-2 infection with real-time polymerase chain reaction (rRT-PCR) assay on nasopharyngeal swab and/or broncho-alveolar lavage (BAL) specimens, belonging to the COVID-HUS cohort [36] and admitted to the medical and surgical critical care units in Hautepierre Hospital (Strasbourg University Hospital) from March 1 to May 1, 2020 were included.

The study was designed and reported to fulfill the STROBE (Strengthening the Reporting of the Observational studies in Epidemiology) statement [37]. The protocol was approved by the Ethics Committee on March 27, 2020. Data and samples were accessed for research purposes from March 27^th^, 2020 to January 30^th^, 2022. All participants or their next of kin gave written informed consent for research according to protocols approved by the institutional review board of Strasbourg University Hospitals (ClinicalTrials.gov NCT 04405726).

### 2. Procedures

We obtained demographic, epidemiological, clinical, basic laboratory and computed tomography scanner (CT Scan) data from patients medical records. Clinical and laboratory data were collected on the day of admission and every seven days until one month of follow-up. To have a homogeneous population of COVID-19 patients with comparable severity, patients with a hospital length of stay longer than 7 days prior to ICU admission and patients with length of stay in ICU shorter than 14 days (excluding deaths) were excluded from the study. All data were collected by the same physician (NP).

#### 2.1. Demographic and epidemiological data collection.

Data on age, gender, body mass index (BMI, with obesity defined by a BMI > 30 kg/m^2^), comorbidities (heart failure, chronic kidney failure, chronic respiratory diseases, active or previous cancer, hypertension, smoking status, type 1 or type 2 diabetes) were collected.

#### 2.2. Clinical data collection.

As per protocol in our institution and for severe COVID-19, patients were mechanically ventilated with tidal volume set to 6 mL/kg ideal body weight, respiratory rate initially set to avoid lung hyperinflation (end expiratory flow equal to 0) and then steered according to blood gas analysis and PEEP set to minimize driving pressure (kept inferior to 14 cmH_2_O) and to maintain plateau pressure under 30 cm H_2_O. Data on ventilatory parameters (i.e., tidal volume [Vt], plateau pressure [Pplat], positive end-expiratory pressure [PEEP], FiO_2_ and PaO_2_/FiO_2_ ratio) were collected. Derived measured variables were calculated as follows: the driving pressure (DP = Pplat – PEEP expressed in cmH_2_O) and the static lung compliance (Cstat = (Vt/DP) expressed in mL/cmH_2_O). We also recorded the use of corticosteroids. Finally, to assess the severity of patients on admission, the SAPS II [38] and SOFA [39] scores were collected.

#### 2.3. Laboratory data collection.

On the day of admission and every seven days, measurement of IL-6 and KL-6 blood concentration were collected. At the same time, we also collected white blood cells, lymphocytes, red blood cells, C-reactive protein, D-dimers, aspartate transaminase (AST), alanine transaminase (ALT), and creatinine blood concentrations. IL-6 and KL-6 were recovered from the COVID-HUS serum library. Serum samples were collected in serum separator tubes, centrifuged, and stored at -20°C until evaluation. IL-6 blood concentration was measured using a new generation ELISA method from Protein Simple® and adapted on the ELLA® machine (BioTechne San José®, California 95134, USA). KL-6 blood concentration was measured by the CLEIA technique using the Lumipulse G1200® (FUJIREBIO Diagnostics Inc®, Courtaboeuf, France).

#### 2.4. CT-Scan data collection.

CT-Scan data were retrieved from scans performed at the Hautepierre Hospital, near the date of ICU admission and every seven days during the hospital stay until day 28. All examinations were acquired from 64-rows (or more) scanners, with a voltage ranging from 80 to 120 kilovolts (kV) depending on the patient’s morphotype. Patients were always placed in the supine position, with their arms raised above head whenever possible. Intravenous iodinated contrast was used to perform pulmonary angiography when pulmonary embolism was suspected. Lung parenchyma was reconstructed using millimeter slices.

Semi-automatic and automatic lung volume measurements were performed using the VP-Lab® software (Visible Patient®, Strasbourg, France). The semi-automated method described in the study by Noll *et al* [40] provides a segmentation of lung volumes into 4 zones:

- “non-aerated” for completely non-aerated lung zones (condensation, atelectasis);
- “poorly-aerated” for imperfectly ventilated lung zones (incomplete alveolar air filling);
- “overinflated” for lung areas corresponding to emphysema and/or overdistension; and
- “normally aerated” segmented into “ground-glass” and “normally restricted” corresponding to ground-glass lung areas and healthy lung areas.

The automatic method allows measurement of 3 types of lung volumes; namely, “ground-glass”, “restricted normally aerated”, and “overinflated”.

**Fig 1:**
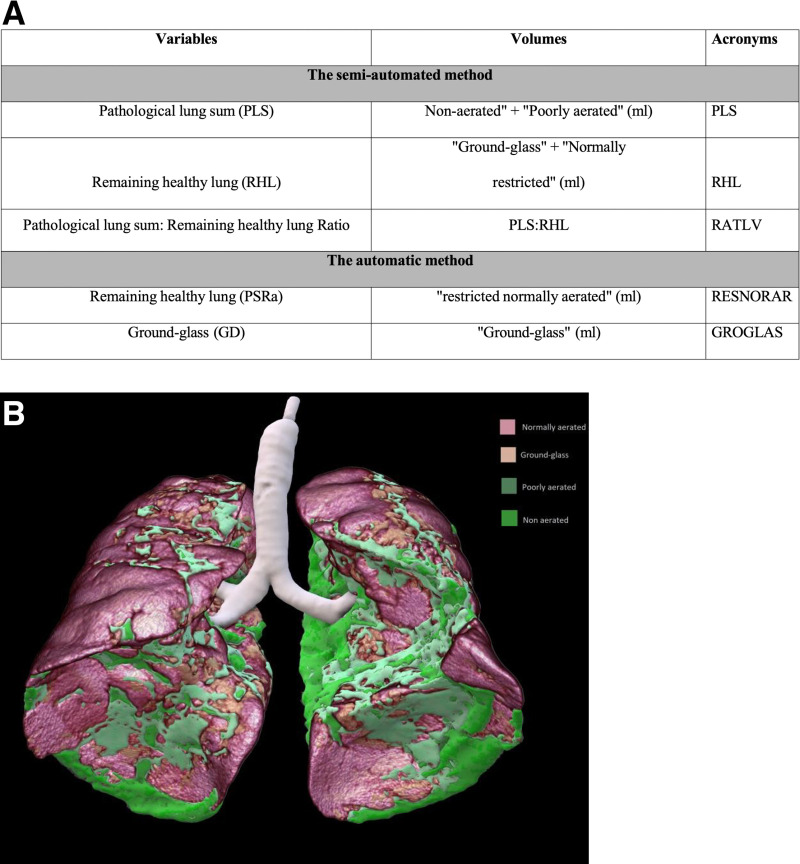
a Lung variables and corresponding volumes. Summary of all pulmonary variables collected and its corresponding volumes (mL), depending on the type of method (either semi-automatic or automatic) used. **b** The different lung volumes.

The variables collected and collated for our study are summarized in **Figs 1a** and b.

### 3. Study endpoints.

The primary endpoint was to challenge the ability of a biological feature of interest (the IL-6/KL-6 ratio), to adequately identify and discriminate three different phenotypes (H, I and L) of COVID-19 ARDS characterized by clinical, spirometric and classical biologic criteria:

1) L phenotype with static lung compliance (Cstat) ≥ 50 ml/cmH_2_O and a systemic inflammatory profile.
2) Intermediate phenotype [10] (I), with a Cstat between 40 ml/cmH_2_O and 50 ml/cmH_2_O.
3) Phenotype H with a Cstat < 40 ml/cmH_2_O and a less systemic inflammatory cytokine profile.

An early, straightforward and accurate distinction of the three ARDS phenotypes would allow a better characterization of COVID-19 ARDS in ICU patients to tailor a personalized therapeutic approach (lung recruitment, tidal volume, PEEP titration, prone positioning).

Secondary endpoints consisted of the following:

a) Assessing the discriminative ability of plasma IL-6 concentrations to differentiate the 3 phenotypes of COVID-19 ARDS.
b) Assessing the discriminative ability of plasma KL-6 concentrations to differentiate the 3 phenotypes of COVID-19 ARDS.
c) Assessing the prognostic performance of the IL-6/KL-6 ratio to determine the risk of death in patients with COVID-19 ARDS
d) Assessing the prognostic performance of IL-6 to determine the risk of death in patients with COVID-19 ARDS
e) Assessing the prognostic performance of KL-6 to determine the risk of death in patients with COVID-19 ARDS
f) Assessing the potential association of IL-6, KL-6 and their ratio with commonly used markers of ARDS severity (compliance of the respiratory system and PaO_2_/FiO_2_ ratio)
g) Assessing the discriminative ability of the IL-6/KL-6 ratio and CT quantification of remaining healthy lung to reflect the severity of lung involvement in COVID-19 ARDS based on compliance of the respiratory system and PaO_2_/FiO_2_ ratio

### 4. Statistical analyses

Categorical variables were described using frequency (n) and proportion (%). Quantitative variables were described using means, medians (m) and interquartile range (IQR).

For the univariate analysis we used a linear mixed model with a random subject effect to account for repeated measures over time [41]. The main effect of each variable on response was modelled using B-splines [42] and where the experimental design allowed, an interaction term between the variable and time was added to the model.

When significant effects were found at the 5% level, post-hoc tests were performed using Tukey’s multiple tests [43].

Note that for all biological data of interest, a logarithmic transformation was performed to reduce the skewness of the data to obtain a better fit to Gaussian models.

To test the association between logIL-6, logKL-6 and the log of their ratio on the one hand and lowest PaO_2_/FiO_2_ and lowest compliance on the other hand, three covariance analysis models were adjusted to study the potential links between the worst PaO_2_/FiO_2_ ratio (PaO_2_/FiO_2_w), the worst compliance (COMPLw) the dichotomous variable (death or survival [D/S]) and each of the variables logIL-6, logKL-6 or logIL-6/KL-6. More specifically, PaO_2_/FiO_2_w and COMPLw were introduced as a response in each of these covariance analysis models, and the D/S was introduced as an explanatory factor. Each of the covariates logIL-6 or logKL-6 or logIL-6/KL-6 was introduced separately as a covariate in the covariance analysis models and three models could thus be adjusted to the data. We carried out permutation tests with the lmPerm package of the R statistical software. This choice of a linear link to model PaO_2_/FiO_2_w and COMPLw was supported by the D/S-wise panel plots of the data.

The multivariate analysis was based on the use of principal component analysis (PCA) [44] implemented in the FactoMineR package [45]. It was performed on both raw and Winsorized data since Winsorzied data are more robust and limit the effect of extreme values. For the multivariate analysis, the dataset had to be imputed with the MissMDA technique [46–48] which is recommended in this situation because MissMDA imputes missing values so the imputed values have no influence on results of the factor analysis. In order to account for the possible effect of extreme values in the PCA, we applied PCA to both raw data and Winsorized data [49]. Winsorized data were derived using the DescTools package [50].

For all tests, the significance level was set at 5%. Effect plots [51] based on estimated marginal means (also called least-squares means or predicted marginal means) [52,53] were drawn using the sjPlot package [54]. These effects plots convey important information about effect size and precision of estimates.

All statistical analyses were performed using R Core Team (2021) [55]

## Results

### Included population

**Fig 2:**
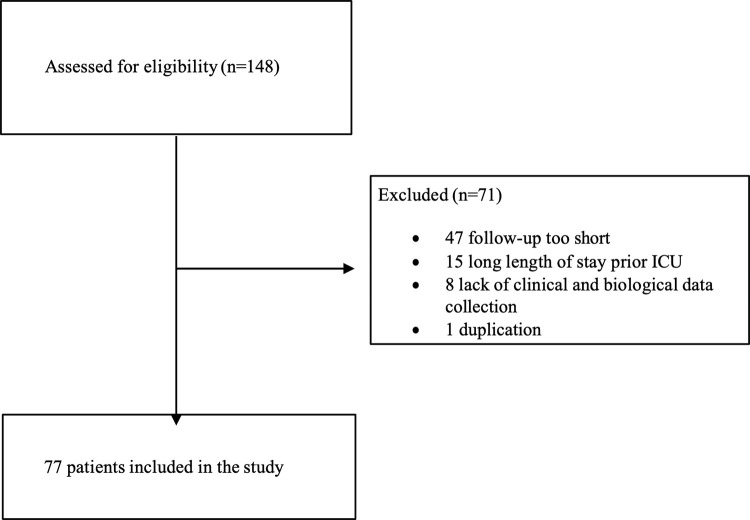
Study Flowchart. The baseline characteristics of the study cohort patients are presented in Tables 1–3.

Among the 148 patients from the COVID-HUS cohort admitted to the ICU and assessed for eligibility between March 2020 and May 2020, 10% were excluded due to a length of stay prior to ICU of more than 7 days and 31% were excluded because of a short follow-up period. Finally, 52% were included in the study (**Fig 2**).

**Table 1:** **Characteristics of the study population.** Categorical variables were described using frequency (n) and proportion (%). Quantitative variables were described using medians (m) and interquartile range (IQR).

Variables	Patients (n = 77)
**Age, years**	65 [58–73]
**Age** ≥ **65 years, n (%)**	44 (57%)
**Sex**	
Male, n (%)	54 (70%)
Female, n (%)	23 (30%)
**Comorbidities**	
Obesity, n(%)	39 (51%)
Heart disease, n (%)	11 (14%)
Chronic renal failure, n (%)	15 (20%)
Chronic respiratory diseases, n (%)	11 (14%)
Malignant tumor, n (%)	21 (27%)
Hypertension, n (%)	46 (60%)
Smoking, n (%)	14 (18%)
Diabetes, n (%)	25 (33%)
**Therapeutics during the ICU stay**	
Mechanical ventilation, n (%)	77 (100%)
Vasopressors, n (%)	77 (100%)
Prone Positioning, n (%)	64 (84%)
Corticosteroid therapy, n (%)	43 (56%)
Hydroxychloroquine, n (%)	47 (61%)
Antiviral, n (%)	19 (25%)
ECMO, n (%)	4 (5%)
**Severity score (on admission to the ICU)**	
SAPS II*	48 [42–56]
SOFA**	8 [7–10]
**Laboratory results on admission (normal lab values)**	
Hemoglobin (13–18 g/dl)	11.7 [10.5-13.1]
WBC (4,1–10,5 Giga/L)	8.3 [6.-11.2]
Lymphocytes (1–4 Giga/L)	0.75 [0.51-0.9]
Platelets (150–400 Giga/L)	213 [168-268]
CRP (<4 mg/l)	204 [112-248]
AST (11–34 U/l)	56 [41-86]
ALT (9–59 U/l)	39 [31-60]
Creatinine (64–104 µmol/l)	85.3 [68.8-148.9]
D-dimers (<500 µg/l)	2085 [1100-3790]
IL-6 (pg/ml)	173 [82.3-333]
KL-6 (U/ml)	478 [311-598]

ICU = intensive care unit; ECMO = extra-corporeal membrane oxygenation; WBC = white blood cells; CRP = C-Reactive protein AST = Aspartate transaminase; ALT = Alanine transaminase

* The Simplified Acute Physiology Score II [38]

** Sequential Organ Failure Assessment Score [39]

**Table 2:** Spirometric characteristics. Qualitative variables were described using frequency (n) and proportion (%). Quantitative variables were described using medians (m) and interquartile range (IQR). In one patient, the static compliance of the respiratory system could not be defined due to instability of the plateau pressure and in two other patients, the measure was considered not close enough to the intubation period.

Variables	Patients (n = 74)	Missing data
**Spirometry (on admission in ICU)**		3
Static compliance (mL/cmH_2_O)	34.5 [25.4-43.4]	
**Spirometry by phenotype (on admission in ICU)**		3
L Phenotype	11 (15%)	
I Phenotype	15 (20%)	
H Phenotype	48 (65%)	
**Oxygenation parameter (on admission in ICU)**		0
PaO_2_/FiO_2_	158 [128-209]	

**Table 3:** Time course of COVID-19 and Outcomes. Qualitative variables were described using frequency (n) and proportion (%). Quantitative variables were described using medians (m) and interquartile range (IQR).

Variables	Day	Missing data
Duration of symptoms before hospitalization	7 [3–9]	0
Length of hospital stay before ICU admission	1 [0-3]	0
**Length of hospital stay**		
ICU length of stay	22 [12-29]	0
Total hospital length of stay	34 [14-51]	5
**Outcomes**		
Mechanical ventilation duration	15 [9-26]	4
Vital status D28	37 (48%)	12
Ventilator-free days	2 [0-2]	11
Death in ICU	30 (39%)	0

Ten patients were not intubated at the time of CT scan. In those patients, a median of 5 days was observed between CT scan and data collection. Almost half of the patients died while in the ICU. Of these patients, 9 died of refractory hypoxemia, 4 of pulmonary embolism, 14 of refractory shock, 2 of treatment limitation, and 1 of hemorrhagic shock. The different phenotypes (H, I and L) did not display different outcomes (Pearson’s Chi-squared test with simulated p-value [based on 2000 replicates], p = 0.2289).

### IL-6/KL-6 ratio and COVID-19 ARDS phenotypes

**Fig 3:**
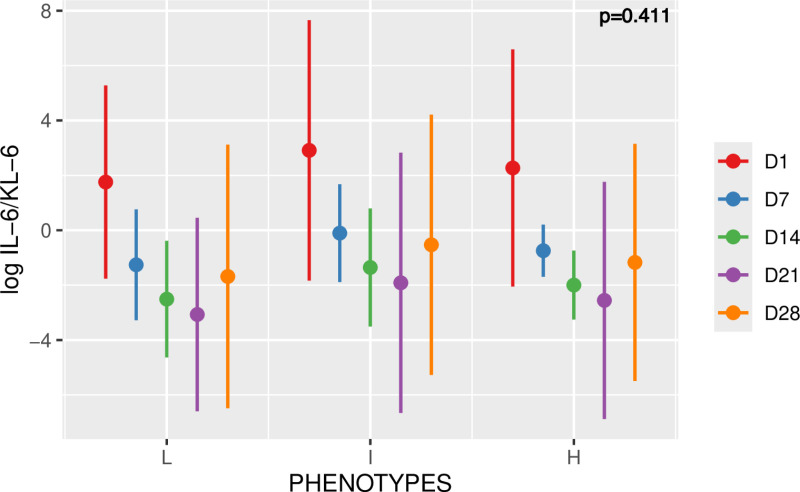
Time course of log IL-6/KL-6 among the L, I and H phenotypes. Values are mean plus/minus standard deviation. Negative values are possible because they are predicted values. For each phenotype, 5 time points were considered (D1, D7, D14, D21, D28). Linear mixed model; p=0.411.

The analysis by linear mixed model showed no significant difference in the mean values of the log ratio IL-6/KL-6 between the different phenotypes. The mean predicted value for the L, I and H phenotypes were -1.45 (±3.21), -0.22 (±3.52), and -0.85 (±2.9) respectively. Moreover, as depicted in **Fig 3**, the IL-6/KL-6 ratio evolved according to similar kinetics over time between the 3 phenotypes without significant difference. This kinetic of the log ratio IL-6/KL-6 was decreasing, for the 3 phenotypes except at day 28.

### IL-6/KL-6 ratio and COVID-19 ARDS outcome

**Fig 4:**
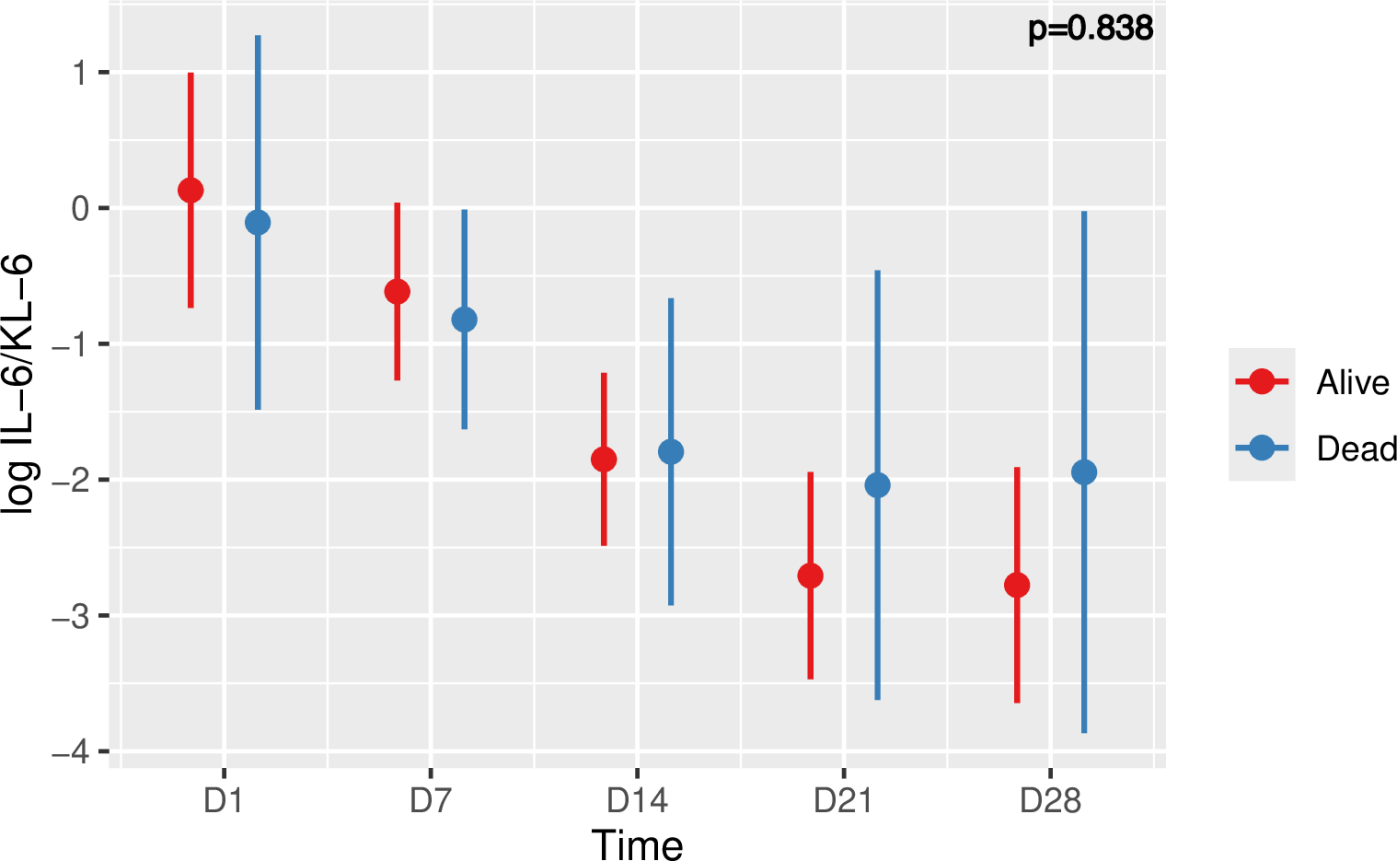
Time course of log IL-6/KL-6 and risk of death in patients with COVID-19 ARDS. Values are means plus/minus standard deviations. Negative values are possible because they are predicted values. Five time points were considered (D1, D7, D14, D21, D28). Linear mixed model, p=0.838.

There was a greater decrease in the log IL-6/KL-6 ratio in surviving patients (from an estimated mean value of 0.13 (±0.43) at day 1 to an estimated mean value of -2, 78 (±0.43) at day 28), compared to deceased patients in whom the log IL-6/KL-6 ratio changed from an estimated mean value of -0.11 (±0.68) at day 1 to an estimated mean value of -1.95 (±0.95) at day 28 (**Fig 4**). Even though the evolution of the log IL-6/KL-6 ratio seemed to be different over time between surviving and deceased patients, the analysis by mixed linear model did not reveal any statistically significant difference.

### IL-6 and COVID-19 ARDS phenotypes

**Fig 5:**
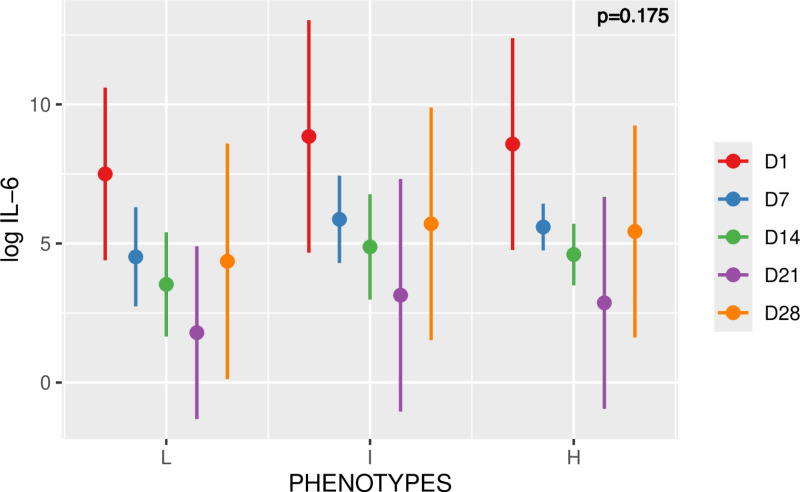
Time course of log IL-6 over time among the L, I and H phenotypes. Values are means plus/minus standard deviations. Negative values are possible because they are predicted values. For each phenotype, 5 time points were considered (D1, D7, D14, D21, D28). Linear mixed model, p=0.175.

We observed similar levels of log IL-6 between the different phenotypes with a predicted mean value for the L, I, and H phenotypes of 4.4 (±2.74), 5.47 (±3.19), 5.4 (±2.56), respectively (**Fig 5**). The analysis by linear mixed model did not reveal any statistically significant difference between phenotypes.

### IL-6 and COVID-19 ARDS outcome

**Fig 6:**
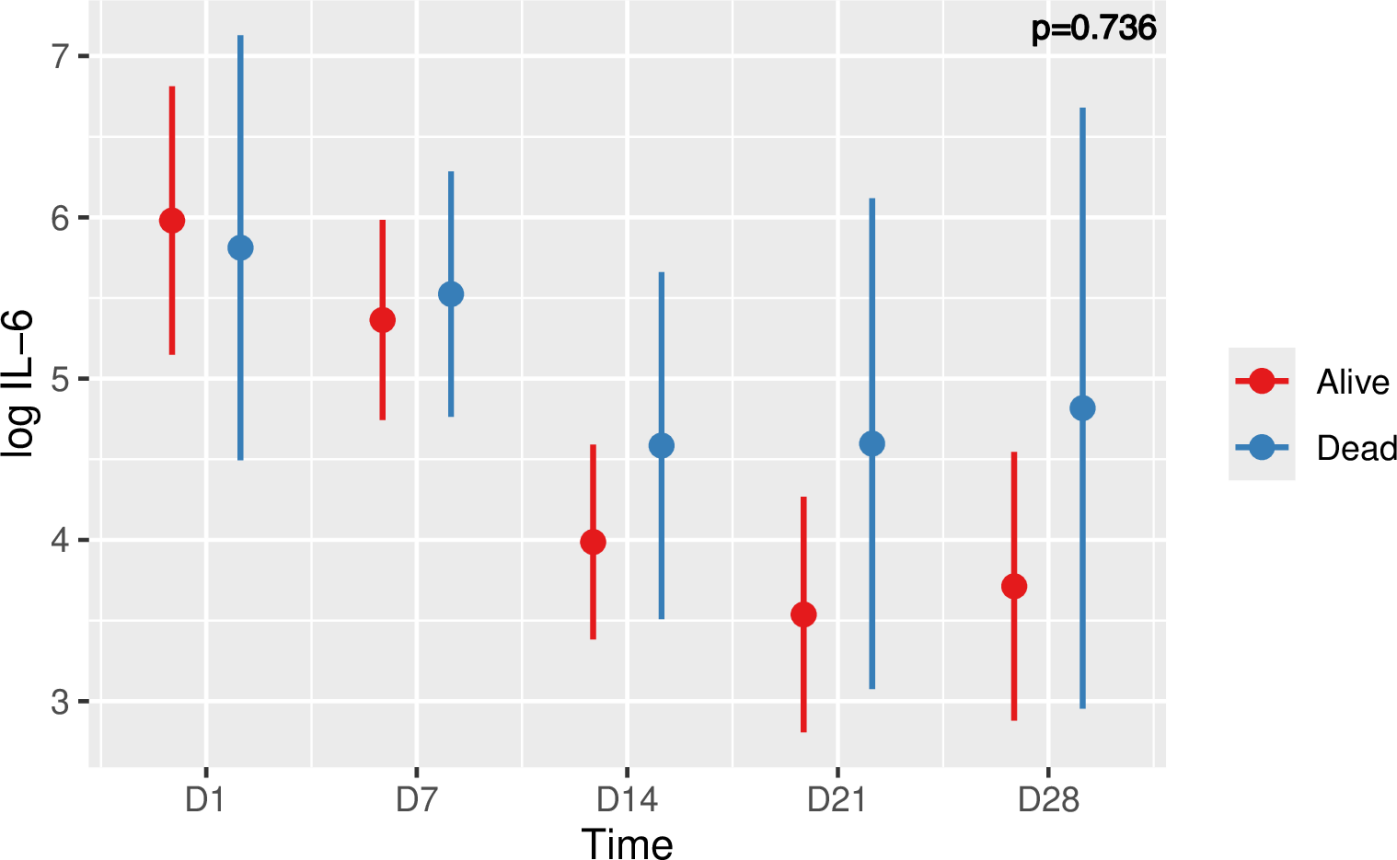
Time course of Log IL-6 and risk of death in patients with COVID-19 ARDS. Values are means plus/minus standard deviations. Five time points were considered (D1, D7, D14, D21, D28). Linear mixed model, p=0.736.

Concerning the possible prognostic properties of the IL-6 in determining the risk of death in patients with CARDS, it can be seen from **Fig 6**, that there was a constant decrease in surviving patients (from an estimated mean value of 5.98 (± 0.41) at day 1 to an estimated mean value of 3.71 (±0.41) at day 28), whereas in deceased patients, there was a less marked decrease (from an estimated mean value of 5.81 (± 0.65) at day 1 to an estimated mean value of 4.82 (±0.92) at day 28). It should be noted that the mean log IL-6 level in the deceased patients already tended to reach a plateau from day 14 with an estimated mean value of 4.59 (±0.53).

Analysis by linear mixed model detected a statistically significant difference for all means over time. The post-hoc analysis with Tukey’s test revealed a statistically significant difference with a p < 0.0027 in the kinetics of the ratio for living patients between time 1 (day 1) and time 5 (day 28).

Despite this evolution of log IL-6 which seemed to be different over time between living and deceased patients, we did not detect a statistically significant difference via the linear mixed model analysis. However, we found a different estimated mean log IL-6 level of 4.52 (±0.16) for living patients compared to 5.07 (±0.30) for deceased patients. Analysis by linear mixed model revealed this difference was statistically significant (p = 0.02).

### KL-6 and COVID-19 ARDS phenotypes

**Fig 7:**
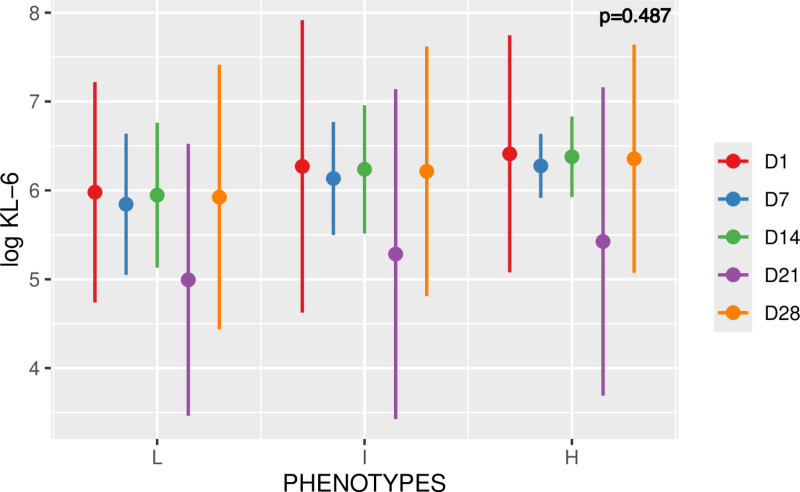
Time course of log KL-6 among the L, I and H phenotypes. Values are means plus/minus standard deviation. Five time points were considered (D1, D7, D14, D21, D28). Linear mixed model, p=0.487.

Linear mixed model analysis showed no significant difference in the mean values of log KL-6 between the different phenotypes (p = 0.487). The average predicted value for the L, I, and H phenotypes were 5.74 (±1.14), 6 (±1.18), and 6.19 (±1.04), respectively. **Fig 7** depicts similar kinetics over time between the 3 phenotypes. This KL-6 kinetic remained unaltered, for the 3 phenotypes except at day 21.

### KL-6 and COVID-19 ARDS outcome

**Fig 8:**
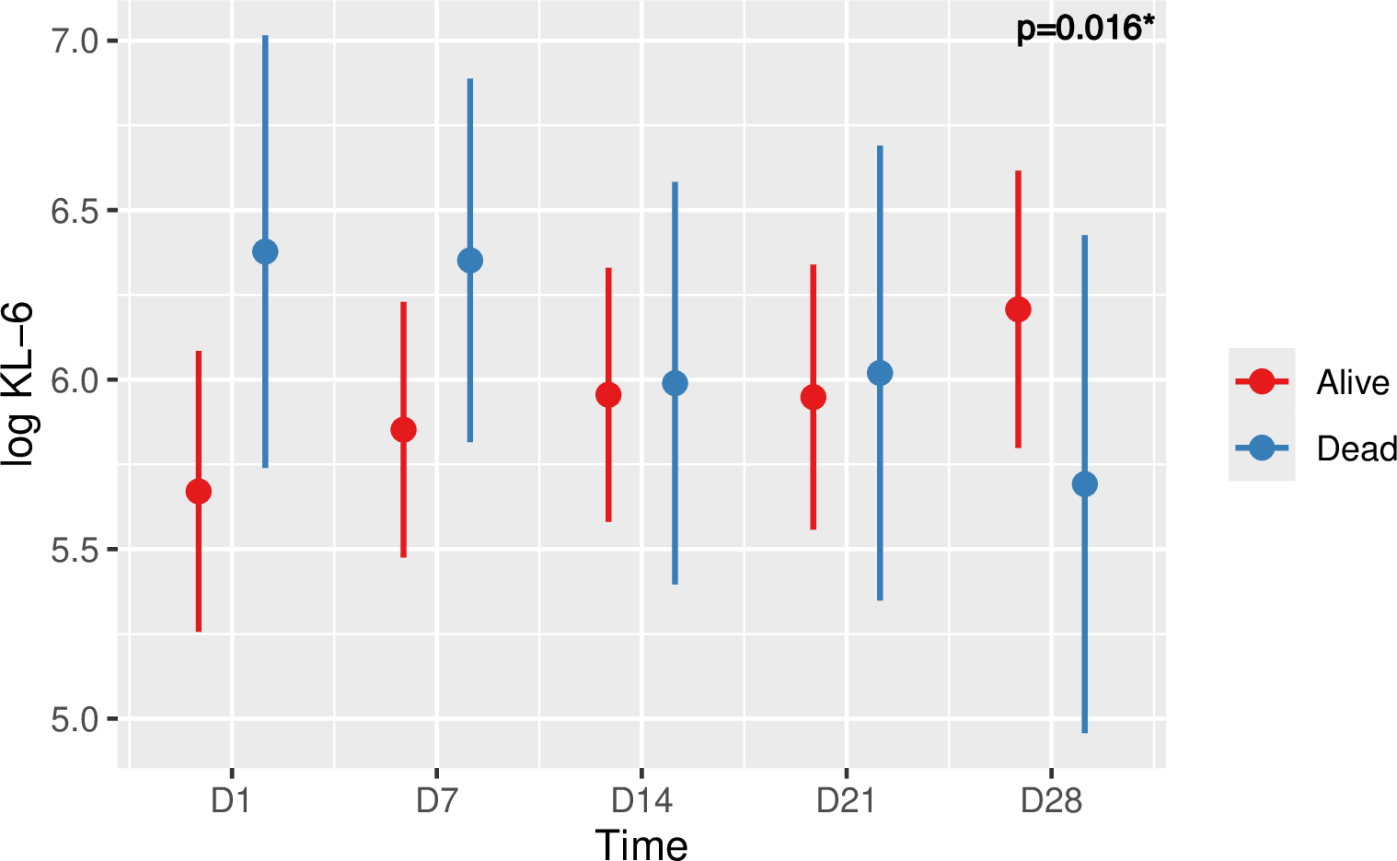
Time course of log KL-6 and risk of death in patients with COVID-19 ARDS. Values are means plus/minus standard deviation. Five time points were considered (D1, D7, D14, D21, D28). Linear mixed model, p=0.016.

Concerning the possible predictive value of IL-6 in determining the risk of death in patients with COVID ARDS, it can be seen from **Fig 8**, that there was a constant increase in living patients over time with an estimated average rate rising from 5.67 (±0.21) at day 1 to 6.21 (±0.20) at day 28, whereas in the deceased, there was a constant decrease from 6.38 (±0.32) at day 1 to 5.69 (±0.36) at day 28. The curves cross at D14. This different evolution over time between the living and the dead was statistically significant via analysis by linear mixed model (p = 0.016).

### Association of IL-6, KL-6 and their ratio with markers of ARDS severity

No significant association was found between logIL-6 and either worst PaO_**2**_/FiO_**2**_ ratio or worst compliance.

LogKL-6 was significantly associated with the worst PaO_2_/FiO_2_ ratio (linear regression [covariance analysis] with permutation tests; p = 0.0219), even considering the Death/Survival status (p = 0.0315).

For the logratio IL-6/KL-6, the linear regression (covariance analysis) approach with permutation tests demonstrated a significant association with worst compliance at the 5% threshold (p = 0.0058). Moreover, it allowed to consider the existence of the dichotomy (Death/Survival), the average level of compliance in both groups being considered significant (p = 0.0042) and the nature of the induced relationship between compliance and logratio IL-6/KL-6 being considered significantly different (p = 0.0056).

### Radio-biological correlates

**Fig 9:**
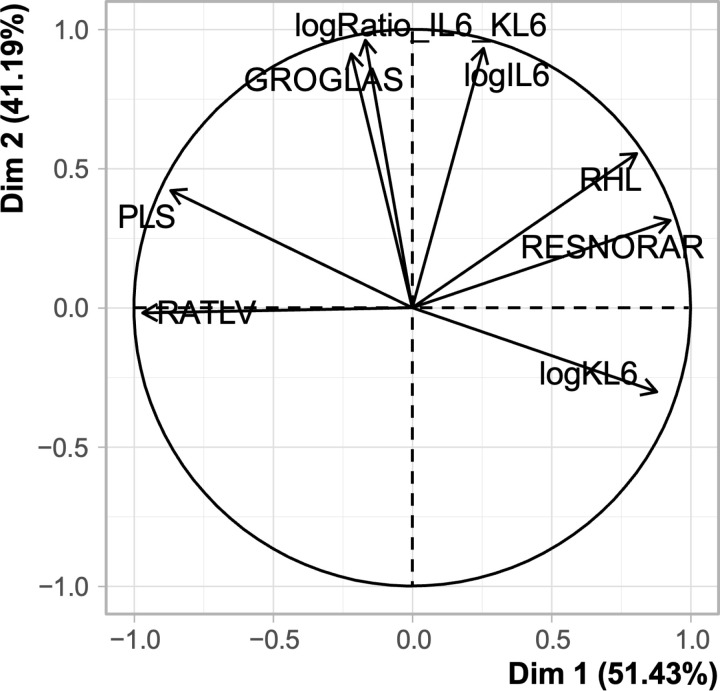
Exploratory PCA on Winsorized data.

Associations between biological (IL-6, KL-6 and their ratio) and CT-derived data (semi-automatic and automatic methods) are shown in **Fig 9**.

This circle of correlations shows 2 main axes (dimensions) explaining 92% of the variance of patients. Each vector is a variable. There are 8 variables of interest, taken on admission: logIL-6, log KL-6, log IL-6/KL-6 ratio, Ground-glass (GROGLAS), Remaining healthy lung (RESNORAR) from the semi-automated method, Remaining healthy lung (RHL) from the automatic method, Pathological lung sum (PLS) and ratio of pathological lung to healthy lung (RATLV).

The results of the principal component analysis (PCA) on raw and Winsorized data were similar. Our PCA had two main components, so two main axes, dimension 1 (DIM 1) and dimension 2 (DIM 2), which explained 92% of the variance of the individuals, resulting in a small loss of information. All the variables in the PCA were well represented on the circle of correlations, which are all close to 1. For easier reading, refer to Fig 1 for the abbreviations of each lung volume.

According to our PCA, there was a linear correlation between IL-6 and ground glass volume (GROGLAS), which evolved in the same direction. Similarly, there was an association between IL-6 and the remaining healthy lung volume (RHL), with a similar evolution but looser association. Note that IL-6 and the “restricted normally aerated” volume (RESNORAR) were not correlated. Similarly, PCA revealed no correlation between IL-6 and the sum of pathological lung (PLS). The IL-6/KL-6 ratio evolved similarly to IL-6 regarding GROGLAS. There was a strong association between the IL-6/KL-6 ratio and GROGLAS, closer than that between IL-6 and GROGLAS. There was no correlation between the IL-6/KL-6 ratio and the RHL or RESNORAR. The correlation between the IL-6/KL-6 ratio and the PLS was minimal. For KL-6, there was a negative linear correlation between KL-6 and the PLS, which evolved in opposite directions. In the same way we noted a loose association between KL-6 and GROGLAS, with an evolution in opposite directions. Moreover, there was a positive linear correlation between KL-6 and the RESNORAR. We found no significant association between CT-derived data (semi-automatic and automatic methods) on the one hand and PaO_2_/FiO_2_ or compliance on the other hand (data not shown).

## Discussion

Demographically, we had a typical population of patients with severe COVID-19, hospitalized in the ICU presenting with similar characteristics as in previous studies [23,56–57]. Thus, this was a predominantly male, middle-aged, obese population with multiple comorbidities including hypertension and diabetes.

Based on median SOFA and SAPS II scores on admission (8 and 48, respectively), our cohort gathered a sample of very seriously ill COVID patients. In comparison, the study by Wang et al [56]. found a median SOFA of 5 score for patients in the ICU, as did the larger COVID-ICU group [3] study with severe SARS-CoV-2, which also found a mean SOFA of 5 and a mean SAPS II score of 37. To further support the severity of our cohort, the median duration of mechanical ventilation was 15 days, ventilator-free time at day 28 was 2 days, duration of hospitalization in the ICU was 22 days with mortality rate in the ICU of 39%. In comparison, in the COVID-ICU group study [3], the duration of mechanical ventilation was 12 days, duration of hospitalization in the ICU was 16 days, and the ICU mortality rate was 35%.

This increased severity in our cohort could be explained by the fact that the Hautepierre Hospital is one of the two reference centers in the Alsace region. As a result, the most severe cases with high comorbidities were referred to this center, especially during this first epidemic wave when the Grand-Est region was severely affected with peripheral hospitals overwhelmed by the surge of COVID-19 cases, especially in the ICU. In addition, for the sake of bed availabilities, the most stable patients were selected daily to be transferred to regions less affected than Alsace by the COVID-19 pandemic [58], resulting in an overrepresentation of patients with hypertension, diabetes, chronic renal failure, chronic respiratory disease or a history of cancer, in proportions greater than those published in the literature [3,56,57]. Despite this severity, at admission patients had moderate level of hypoxemia with a median PaO_2_/FiO_2_ ratio of 158, similar to the COVID-ICU group [3]. Static compliance on admission was 34.4 ml/cmH_2_O, which is far from the almost “normal” compliance (median 50 ml/cmH_2_O) reported by Chiumello et al. [59] in 32 patients with severe COVID-19; however, this compliance value is like those reported in many studies [3,60] with much larger cohorts of COVID-19 patients. The severity of our cohort and the fact that we had a cohort with a highly inflammatory state can be seen in the biological characteristics of our patients, and in particular the biological variables that have been retained as prognostic factors for the severity of COVID-19, i.e., D-dimer levels [61], CRP [62] and lymphopenia.

Even if the association of these variables with the severity of SARS-CoV-2 infection has been demonstrated, their optimal cut-offs allowing the accurate prediction of patient outcome with satisfying sensitivity and specificity remain to be determined. Thus, the median D-dimer value of 2085 μg/ml is well above that found in the COVID-ICU group [3] cohort, of 1600 μg/ml.

Similarly, the median CRP concentration of 204 mg/l is well above the median CRP of 136.3 mg/l found in 990 critically ill patients in the New York study [63] among a cohort of 5279 patients hospitalized with SARS-CoV-2 infection. This significant inflammatory state is well represented by lymphopenia, which was 0.75 Giga/L, similar to that found in the literature [64].

### IL-6 at admission

The median IL-6 level on admission in our cohort was 173 pg/ml corroborating patients’ severity, who presented hyperinflammation and probably a cytokine storm. The level of IL-6 found was much higher than those reported in other studies [65].

These previous studies, reporting lower plasma concentrations of several pro-inflammatory cytokines (including IL-6) in COVID-19 patients verses those with ARDS, cytokine release syndrome or in septic patients, have cast doubt on the very existence of the cytokine storm in COVID-19 for some authors [66].

The higher IL-6 levels in our study compared to those usually found in cytokine storm may be explained by the fact that a large proportion of patients in previously published studies had moderate disease severity which may have minimized the mean IL-6 titers. Moreover, the recently published study by Gorham et al. [67], which assessed the outcome of patients with IL-6 levels measured repeatedly during COVID-19 in patients admitted to the ICU, found much higher levels, like those in patients with hypoinflammatory ARDS [66]. We also have excluded from our analysis COVID patients whose duration of ICU stay was below 14 days. This probably has excluded many “less severe” (and possibly less inflamed) patients and may also explain the higher pro-inflammatory cytokines reported in this compared to other ones.

Another factor that may explain the low levels of IL-6 reported in other studies relies on the paucity of measurements, mainly at admission [57], which neither reflects the inflammatory peak, nor the evolution of inflammation in patients with SARS-CoV-2, particularly those with moderate forms.

Finally, there was real clinical improvement over time in patients on corticosteroids or anti-IL-6 therapies. However, a change in therapeutic strategies during the first epidemic wave of COVID-19 due to the rapid acquisition of new knowledge, may have modified the IL-6 levels of the various cohorts, with a possible decrease after the widespread use of corticosteroids or anti-IL6. Thus, initially, some patient cohorts were treated with antibiotics systematically, some with antivirals such as Ritonavir, while others used corticosteroids from the beginning of the epidemic wave. In our cohort, these changes in treatment also took place, as shown by the proportions of patients treated with corticosteroids, hydroxychloroquine, and antivirals.

### KL-6 at admission

The median KL-6 level on admission was 478 U/ml, which is in the same order of magnitude as that found by Frix et al. [68] with a median level of 405 U/ml; however, this study involved patients with COVID-19 who were hospitalized without distinction between severe and moderate forms. The study by Deng et al. [28] suggests a median level of 898 U/ml in patients in a severe state, which is much higher than what was found in our study but encompasses the entire hospital stay.

The comparison of these two studies with ours is very interesting. It suggests that our patients were not at a very advanced stage in the evolution of COVID-19 when they were first sampled for KL-6 assay. This is also suggested by the short duration of evolution (approximately 1 week) between the onset of symptoms and hospitalization in intensive care. Conversely, as highlighted in the study by Deng et al. [28], the peak of KL-6 is situated for patients with a severe form of SARS-CoV-2 around the third week after the onset of symptoms, which probably explains the level of 405 U/ml at admission in our cohort.

### Phenotypes classification and IL-6/KL-6 ratio

According to our results, the proportion of patients with a H phenotype on admission was 65% compared to 15% of patients with an L phenotype and 20% with the intermediate I phenotype. In the paper by Chiumello et al. [59], which sets the stage for a relatively normal compliance in patients with severe COVID-19 compared to other ARDS patient populations, there were 34% of patients with a H phenotype and 47% with an L phenotype on admission. Our asymmetry in proportions may have led to a loss of comparability between the L and H phenotypes because of limited data for L phenotype patients. Comparing the median duration of symptom evolution until hospitalization, which is similar to what is found in the literature [57], and the median duration of hospitalization prior to ICU admission, we find a difference of 6 days. Given that after one week of symptom evolution, a proportion of patients are already evolving towards an H phenotype, our results suggest that a large proportion of our cohort were either in transition or already in an H phenotype.

Our choice of study entry date, which corresponded to the date of admission to the hospital, may therefore have been too late. The challenge of taking patients at the onset of symptomatology lies in the retrospective nature of our study in which the date of symptoms onset was declarative and early important spirometric and biological data unavailable.

Another important limitation is the small number of patients included in our study, leading to an asymmetry in the proportions of patients per phenotype. A solution to prevent this asymmetry would have been to include patients with a shorter duration of follow-up in the ICU. This would have allowed the inclusion of 47 additional patients in our cohort. However, the risk of having too much missing data guided our choice to exclude them from analysis.

Yet, in view of the overall results concerning the IL-6/KL-6 ratio, IL-6 and KL-6 concentrations, which seem to have similar levels across the different phenotypes without statistical significance, one may wonder whether these elements are simply not discriminating enough to distinguish the phenotypes, or whether we lacked statistical power. Gatinnoni et al. [9] also concede that the COVID-19 ARDS phenotypes are temporally dynamic, not mutually exclusive, and therefore there may be overlap between them. This was also recently demonstrated to hold true in other non-COVID ARDS.[69]

We may also wonder whether our study, which combines a single spirometric parameter (compliance, proposed in Gatinnoni’s classification [9]) with biological parameters, is sufficiently discriminative to decipher the phenotypes previously described. It is likely that the addition of supplementary parameters, and in particular CT scan analysis, would have enabled us to refine our results.

### Prognostic and IL-6, KL-6, ratio IL-6/KL-6

One of the hypotheses of our study concerned the possible prognostic character of the IL-6/KL-6 ratio.

For IL-6, in our severe ICU cohort, we were able to demonstrate a statistically significant difference in mean levels between survivors and deceased patients over the first 28 days, which supports what has already been shown in the literature [67,70]; however, we did not show a difference in the time course of IL-6 levels over time between living and deceased patients. This difference in evolution, particularly between the 2^nd^ and 3^rd^ week after the onset of symptoms in COVID-19 patients, has already been shown in the literature [24,67]. The hypotheses that may underpin this lack of difference in relation to the literature lie in the type of population under study. For example, the study by Zhou *et al.* [24] assessed a population of patients with moderate to critical illness, and the mean IL-6 level may have been minimized over time in the survivors, compared with our cohort of more homogeneous severe ICU patients with much higher IL-6 levels even in the survivors. On the other hand, the number of patients in our cohort may not have been large enough to demonstrate such a difference. Furthermore, there was a significant loss of data after day 14 in the group of patients who died, due to this unfavorable evolution from day 15. It should be noted that the peak of IL-6 for deceased patients and survivors in our study was at the first follow-up timepoint which corresponds to a median of 8 days after the onset of symptoms. For survivors, this is consistent with what has been described in the literature. For instance, Santa Cruz *et al.* [71] also found a peak of IL-6 in survivors between 7 and 10 days after the onset of symptoms. For deceased patients, the literature also shows an initial peak with a decreasing evolution of IL-6 over time [24,72]. This notion of an initial peak is important in explaining the evolution of the IL-6/KL-6 ratio, which we will develop later.

Regarding KL-6, a very interesting result in our study is the inverse and statistically significant evolution of the mean KL-6 level during the 28 days of follow-up between the survivors and the deceased patients. Thus, for the deceased patients, we describe an initial peak with subsequent decrease. For the survivors, there was an increasing trend without a peak after 28 days of follow-up, which corresponds to about 1 month of evolution, as shown in the study by Deng *et al.* [28]. This is the first study showing this evolution between survivors and decedents in a cohort of ICU patients with COVID-19 ARDS. Previously Kondo *et al.* [73] compared KL-6 levels in ARDS patients over time between survivors and deceased patients. This study did not reveal any difference in the evolution of KL-6 levels in serum between these two populations, but it was found in bronchoalveolar fluid. It should be noted that in our study we did not find any difference in mean KL-6 between survivors and decedents, understandably so because of an inverse evolution of mean KL-6 levels over time with similar orders of magnitude.

The pathophysiological hypothesis of this inverse evolution is interesting. KL-6, which is a prognostic factor of severity in ARDS, interstitial lung disease and in COVID-19, is released by type II pneumocytes and in particular in greater quantities when there is damage to the alveolar-capillary membrane or in the process of regeneration linked to hyperplasia [74–76]. This seems to explain the initial KL-6 level in survivors, with lower levels than in deceased patients because these are patients with less severe forms of ARDS [74,77]. Subsequently, due to a mechanism of regeneration of these type II pneumocytes, and hyperplasia, fibrosis may occur in surviving COVID-19 patients [78]. The KL-6 level should therefore increase over time, in analogy to what is found in the follow-up of interstitial lung disease [79,80]. In deceased patients, the KL-6 level is initially high in association with very severe ARDS [81]. Afterwards, and potentially due to the absence of possible regeneration of type II pneumocytes in these patients with a poor outcome, the KL-6 level will progressively decrease.

All this suggests on the one hand that KL-6 is indeed a prognostic biomarker for mortality and on the other hand that it could allow clinicians to follow the fibrotic process in our ICU patients. This pathophysiological explanation remains an extrapolated hypothesis from our exploratory results and the published literature.

Finally, for the IL-6/KL-6 ratio, this is the first time that it has been analyzed as a possible prognostic factor according to living or deceased status. The only element highlighted was a more important kinetic decrease of the ratio for survivors than for deceased patients. This is evidenced by a statistically significant difference in the evolution of the mean log IL-6/KL-6 ratio in survivors between T1 and T5, which was not found in decedents. This decreasing kinetics for both survivors and deceased patients seems to be largely related to the evolution of IL-6; however, no difference in the overall mean or over time between survivors and decedents was found, (unlike IL-6), disqualifying the IL-6/KL-6 ratio as a suitable predictor of mortality.

### Radio-biological correlates

Concerning the possible association between the IL-6/KL-6 ratio and lung damage as assessed by CT, this could be predictive of the severity of lung involvement in COVID-19 like IL-6 [25] or KL-6 [82].

The PCA analysis unveiled a very interesting linear association between the IL-6/KL-6 ratio and ground glass lung volume, an association which was stronger than with IL-6. This suggests that the IL-6/KL-6 ratio may be predictive of the severity of certain typical lung lesions associated with COVID-19 ARDS. While an association between KL-6 blood concentration and lung involvement on semiquantitative CT score was already reported [83], our study reports a stronger correlate of IL-6/KL-6 ratio with ground glass volume in severe ventilated ICU patients. Moreover, it is also the first time that a comparison between two biomarkers is performed to determine which one better predicts the severity of lung lesions. The IL-6/KL-6 value in COVID-19 patients might be valuable to speed-up diagnostic workflow in symptomatic and hypoxemic patients in whom transfer to the CT facility is sometimes risky. Further studies evaluating this association will be necessary to confirm this potential breakthrough.

### Strengths and limitations of the study

Our study had some strengths, which rely on innovative techniques and robust methodology. VP-Lab® software was used as a new tool for measuring lung volumes. This automated quantification is innovative, in a way that it can be used to monitor GROGLAS, which is a marker of the severity damage in COVID-19. Furthermore, static and dynamic modeling approaches were compared in patients with COVID-19-related ARDS [33,84]. These studies revealed the added value of modeling variation as a function of time to understand the heterogeneity of ARDS. The dynamic pattern of COVID-19 ARDS was taken into account in our study with multiple clinical and biological analysis across different time points. Finally, the collaboration with a team of mathematicians with a solid biostatistics background, almost naive to the medical field, allowed the use of unsupervised and supervised Bayesian approaches for the processing of data, bringing a new perspective to what is usually done.

Our study also had some limitations. The main one being its retrospective nature with the inherent biases of this type of study. As a result, a significant amount of data was missing, which could have affected our results despite using a multiple imputation method. Another limitation is the monocentric nature of the study, which led to a selection bias. Third, 48% of our initial cohort was excluded to harmonize our population over time. This may have led to a selection bias that we accept for the sake of data completeness. Fourth, the fast-changing therapeutic regimen over time (discontinuation of choroquine, introduction of corticosteroids and tocilizumab) may have altered the levels of some cytokines, limiting the generalizability of our results. Fifth, the extra-regional transfer of the less severe patients to relieve the surge [58] also led to a strong selection bias. Sixth, the failure to take into account all the elements defining phenotypes according to the classification of Gattinoni *et al.* [9] probably did not make it possible to be more discriminating in identifying the phenotypes described in his study. Seventh, measurement of cytokine levels in COVID-19 has low specificity. According to Liu et al. [21], although cytokines such as IL-6, TNF-α and IFN-γ are often used as biomarkers of inflammation in severe cases of COVID-19, their clinical interpretation remains complex. Indeed, cytokine levels can vary considerably between patients, making it difficult to establish precise thresholds for diagnosing or monitoring disease severity. Furthermore, the cytokine response is not specific to COVID-19, as elevated levels of cytokines can also be observed in other infections or inflammatory conditions. Therefore, this low specificity may reduce the usefulness of cytokine measurements to reliably predict clinical course. In addition, the variation in assay methods and delays in detection of cytokines in serum add to the complexity, limiting their usefulness in rapid and effective clinical decision-making. Measurement of overinflated lung volume would have been of real value with regards to lung compliance and response to PEEP [85]. Unfortunately, we were not able to decipher overdistension from emphysematous lung areas in the “overinflated” category defined by the software. Finally, to test the normality of the model, we had to express our biomarker values in logarithmic form after transformation, resulting in a loss of comparability of our rates with those found in the literature, and requiring us to consider trends only and not raw values.

## Conclusion

This retrospective study is the first to cross-reference a new biomarker (IL-6/KL-6 ratio) with the L, I and H clinico-radiological phenotypes as described in the study by Gattinoni et al. no statistically significant difference was shown in the evolution of the ratio to decipher the 3 phenotypes at any time, and therefore we could not confirm our main hypothesis. The principal component analysis revealed that the log IL-6/KL-6 ratio was linearly related to automatically-determined ground glass volume on lung CT and may therefore be predictive of lung involvement in COVID-19. Other studies combining lung epithelial and endothelial biomarkers with clinical and CT criteria may facilitate the rapid distinction of COVID-19 ARDS phenotypes and pave the way for both ventilatory and drug-based personalized approaches.

## Supporting information

S1 Data Demographic, clinical, spirometric and biological data of the included patient population. (XLSX)
